# Molecular insights into the pathophysiology of doxorubicin-induced cardiotoxicity: a graphical representation

**DOI:** 10.1007/s00204-022-03262-w

**Published:** 2022-03-25

**Authors:** Nonhlakanipho F. Sangweni, Kwazi Gabuza, Barbara Huisamen, Lawrence Mabasa, Derick van Vuuren, Rabia Johnson

**Affiliations:** 1 Biomedical Research and Innovation Platform, South African Medical Research Council, Tygerberg, P.O. Box 19070, Cape Town, 7505 South Africa; 2 Centre for Cardio-Metabolic Research in Africa, Division of Medical Physiology, Faculty of Medicine and Health Sciences, Stellenbosch University, Tygerberg, 7505 South Africa

**Keywords:** Doxorubicin-induced cardiotoxicity, Cell death pathways, Endogenous antioxidants

## Abstract

A breakthrough in oncology research was the discovery of doxorubicin (Dox) in the 1960’s. Unlike other chemotherapy drugs, Dox was determined to have a greater therapeutic index. Since its discovery, Dox has, in part, contributed to the 5–10-year survival increase in cancer patient outcomes. Unfortunately, despite its efficacy, both in adult and pediatric cancers, the clinical significance of Dox is tainted by its adverse side effects, which usually manifest as cardiotoxicity. The issue stems from Dox’s lack of specificity which prevents it from accurately distinguishing between cancer cells and healthy cell lines, like cardiomyocytes. In addition, the high binding affinity of Dox to topoisomerases, which are abundantly found in cancer and cardiac cells in different isoforms, potentiates DNA damage. In both cell lines, Dox induces cytotoxicity by stimulating the production of pro-oxidants whilst inhibiting antioxidant enzymatic activity. Given that the cardiac muscle has an inherently low antioxidant capacity makes it susceptible to oxidative damage thereby, allowing the accumulation of Dox within the myocardium. Subsequently, Dox drives the activation of cell death pathways, such as ferroptosis, necroptosis and apoptosis by triggering numerous cellular responses that have been implicated in diseases. To date, the exact mechanism by which Dox induces the cardiotoxicity remains an aspect of much interest in cardio-oncology research. Hence, the current review summarizes the proposed mechanisms that are associated with the onset and progression of DIC.

## Background

It is well established that the 5–15-year survival rate of most cancer outcomes has radically improved in recent decades (Scott et al. 2018). As a result, numerous procedures and drugs are currently available for cancer treatment, with many more being studied (Linschoten Marijke et al. 2018). Such therapies include systemic drugs like chemotherapy, immunotherapy, and targeted therapy as they may affect the entire body. While others include localized treatments like surgery and radiation therapy, which are used to treat specific areas of the body or tumors. However, in more recent years, epidemiological data have shown that these therapies may induce debilitating long-term side effects, like heart disease, and by this, reduce the life expectancy of cancer survivors (Linschoten Marijke et al. 2018; Scott et al. 2018; Visscher et al. 2011). As such, much effort has been dedicated to better understand the long-term effects of these therapies. Therefore, this review aims to summarize the mechanisms linked to the development of cardiovascular dysfunction ensuing from chemotherapeutic drugs, with a specific focus on doxorubicin (Dox)-induced cardiomyopathy (DIC).

## Doxorubicin-induced oxidative stress

**Fig. 1:**
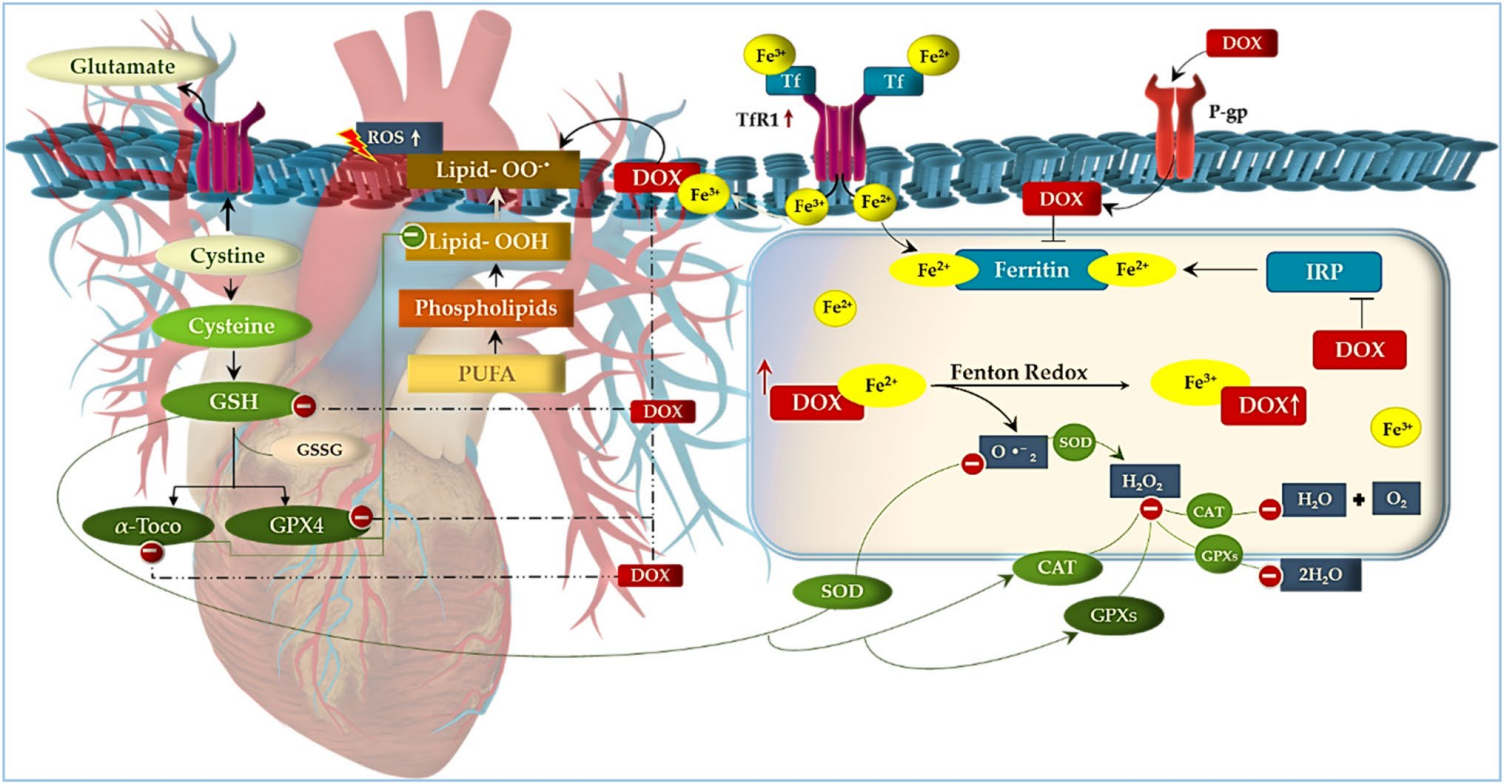
A schematic representation of Doxorubicin (Dox)-induced oxidative stress through cardiac iron overload. Briefly, iron (Fe) overload, by Dox administration, facilitates the formation of Fe–Dox complexes to induce oxidative damage and ferroptosis. Dox’s ability to deplete glutathione (GSH) content, initiates the downregulation of enzymatic (glutathione peroxidases (GPXs), superoxide dismutase’s (SODs), catalases (CATs) and glutathione-S-transferases (GSTs)) and non-enzymatic antioxidant (alpha-tocopherol (vitamin E)), leaving the myocardium susceptible to oxidative tissue damage. This process is further exacerbated by the conversion of hydrogen peroxide (H_2_O_2_), superoxide (O·^–^_2_) and lipid peroxides into more potent ROS. Dox also elevates transferrin (Tf) expression by mutating the human homeostatic iron (HFE) protein, further increasing cardiac Fe-overload and inducing ferroptosis

There are several mechanisms by which the administration of Dox can impair cardiac function, one of which include oxidative stress through iron overload. Generally, the propensity of Dox to disrupt pathways involved in iron metabolism is reported to accelerate iron overload and induce cardiac ferroptosis. These disparities in iron homeostasis accompanied with Dox’s iron-chelating properties, facilitate the formation of iron–Dox (Fe–Dox) complexes which catalyzes the conversion of hydrogen peroxide (H_2_O_2_) to even more reactive oxygen species, such as hydroxyl (OH) radicals (Linschoten et al. 2018; Agunbiade et al*.* 2019). Under normal conditions, these radicals are detoxified by glutathione (GSH), which is an essential reservoir for intracellular cysteine and the most important antioxidant that is pivotal for redox homeostasis (Dukhande et al. 2009). It does this by continuously recycling oxidized biomolecules, i.e., enzymatic, and non-enzymatic antioxidants. The enzymatic antioxidants, which include glutathione peroxidases (GPXs), superoxide dismutase’s (SODs), catalases (CATs) and glutathione-S-transferases (GSTs), are responsible for the detoxification of lipid hydroperoxides, superoxide’s (O·^–^_2_), H_2_O_2_, xenobiotics and endogenous compounds. Additionally, GSH also supports the regeneration of alpha-tocopherol (vitamin E), which is a non-enzymatic fat-soluble antioxidant that prevents lipid peroxidation and stabilizes cell membranes. However, the ability of GSH to transport toxins across the plasma membrane and influence redox activity makes it a suitable target for xenobiotics, like Dox, to drive oxidative damage (Fig. 1).

Doxorubicin is closely associated with GSH depletion through the dysregulation of nicotinamide adenine dinucleotide phosphate (NADPH), which is a critical enzyme that catalyzes the enzymatic conversion of oxidized glutathione (GSSG) to GSH by glutathione reductase (Tanaka et al., 2020; Carrasco et al., 2021). Paradoxically, during Dox treatment, NADPH is recruited and used as a substrate by NADPH oxidases (NOXs) to produce ROS (Xiao et al. 2018). The latter, with the concomitant depletion of GSH, leads to the downregulation of GPX4, α-tocopherol, SOD and CAT, causing oxidative stress. Similarly, the Fe–Dox complexes further exacerbate the oxidative damage by stimulating the conversion O•^–^_2_, H_2_O_2_ and lipid peroxidation into even more potent ROS entities (Linschoten et al. 2018). Additionally, these complexes inactivate the iron regulatory proteins 1 and 2 (IRP1 and IRP2) which triggers iron overload through ferritin inhibition. In the same context, Dox continues to elevate the expression of transferrin (Tf) and its receptor (TfR) by causing mutations in the human homeostatic iron (HFE) protein, permitting more free iron to enter the cardiomyocytes and inducing ferroptosis (Lipschultz et al. 2013; Christidi and Brunham 2021). The intricacy of iron overload, in the failing hearts of cancer patients, is supported by the clinical inclusion of the potent iron-chelating dexrazoxane, which is the only FDA-approved cardioprotective drug for DIC.

## Inflammatory cytokines and necroptosis

**Fig. 2:**
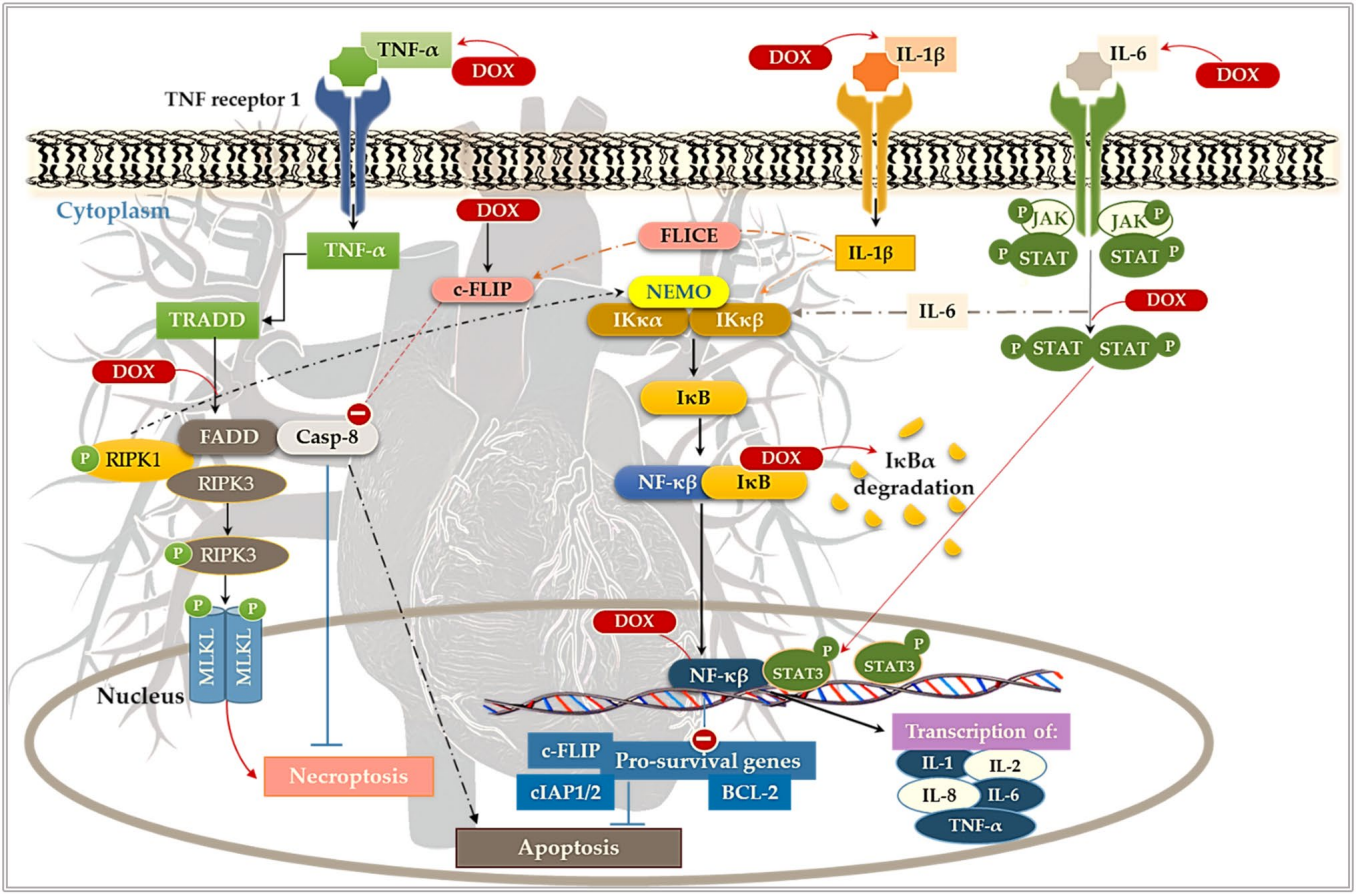
Doxorubicin-induced inflammatory response and resultant. The binding of tumor necrosis factor alpha (TNF-α) to TNFR1 facilitates the assembly of cytoplasmic complex II, consisting of the receptor-interacting protein-1 (RIPK1), caspase-8 (Casp-8) and complex Fas-associated via death domain (FADD). This complex triggers the autophosphorylation and oligomerization of receptor-interacting serine/threonine protein kinases 1/3 (RIPK1 and RIPK3) and the simultaneous inhibition of Casp-8, which initiates necroptosis via the phosphorylation of mixed lineage kinase domain-like protein (MLKL). Additionally, Dox impairs nuclear factor kappa beta (NF-κβ) activity via the overexpression of interleukin 1 beta (IL-1β) and IL-6, and by disrupting the IkappaB (IκB)-kinase (IKK) complex, which is composed of the kinases IKKα and IKKβ and the adaptor protein NEMO (NF-κB essential modulator). Dox inhibition of the Janus kinase (JAK)-signal transducer and activator of transcription (STAT3) pathway impedes the transcription of anti-apoptotic genes and thus, worsens the cardiac outcome

The effects of chemotherapy on pro-inflammatory cytokines, inflammatory cell infiltration and resultant necroptosis are a fundamental aspect of left ventricular dysfunction (LVD), which is a hallmark of DIC (Pecoraro et al. 2016). Necroptosis is a type of regulated cell death that is initiated by specific death receptors, namely Fas and tumor necrosis factor receptor 1 (TNFR1) (Font-Belmonte et al. 2019). Once activated, the death receptors may either stimulate cell survival or death pathways by recruiting specific signaling protein complexes (Font-Belmonte et al. 2019). Concisely, the binding of TNF-α to TNFR1 drives the assembly of a cytosolic caspase-8 (Casp-8) signaling complex (cytoplasmic complex II), which consists of the receptor-interacting protein-1 (RIPK1), Casp-8 and complex Fas-associated via death domain (FADD) (Font-Belmonte et al. 2019) (Fig. 2). Generally, the dual functionality of active Casp-8 enables it to simultaneously initiate apoptosis whilst inactivating essential necroptosis mediators (RIPK1 and RIPK3) (Galluzzi et al. 2018). However, during Dox treatment, the inhibition of the Casp-8 pathway facilitates the autophosphorylation and oligomerization of RIPK1 which in turn phosphorylates RIPK3. Subsequently, RIPK3 drives necroptosis by phosphorylating the mixed lineage kinase domain-like protein (MLKL), which is a pore-forming protein that exposes phosphatidylserine and triggers plasma membrane leakage (Rolski and Błyszczuk 2020; Zhao et al. 2021). In this manner, RIPK1 serves as a critical molecule that not only elicits apoptosis in a Casp-8-dependent manner but also necroptosis via RIPK3 activation.

In contrast, RIPK1 may also prevent the death receptors from inducing apoptosis by activating nuclear factor kappa beta (NF-κβ) (Pescastore et al. 2016). Generally, activation of NF-κβ ensues from the expression of the anti-apoptotic FADD-like interleukin-1β-converting enzyme-like (FLICE) inhibitory protein (cFLIP), which is an inactive homolog of Casp-8 (Galluzzi et al. 2018; Golks et al. 2006) (Fig. 2). Upon activation, NF-κβ may stimulate the transcription of anti-apoptotic genes, namely, B-cell lymphoma-2 (BCL-2), cellular inhibitors of apoptosis (cIAP1 and cIAP2) and cFLIP. Conversely, the prolonged activation of NF-κβ, by Dox exposure, leads to chronic inflammation and resultant heart failure (Proskuriakova et al. 2021; Gordon et al. 2011). Concisely, Dox impairs NF-κβ activity by overexpressing interleukin 1 beta (IL-1β) and IL-6, as well as by disrupting the IκB-kinase (IKK) complex, which is composed of the kinases IKKα and IKKβ and the adaptor protein NEMO (NF-κB essential modulator) and thus, inhibits the transcription of anti-apoptotic genes in the myocardium (Proskuriakova et al. 2021; Barczewski et al. 2019). In the context of myocardial infarction and Dox cardiotoxicity, overexpression of the Janus kinase (JAK)-signal transducer and activator of transcription (STAT3) pathway by protagonists driven by the cytokine IL-6, has been shown to offer cardioprotection (Rong et al. 2016; Fuglesteg et al. 2008; Fuchs et al. 2003). This protection is primarily attributed to the fact that STAT3 is naturally overexpressed in the myocardium but, is further elevated during cardiomyopathy (Harhous et al. 2019; Rong et al. 2016). In contrast, inhibition of the JAK/STAT signaling pathway in cancer cells hampers the expression of genes involved in essential cellular function (cFLIP, BCL-2, Akt, etc.) and prevents tumorigenesis (Bose et al. 2020). Therefore, in this manner, antagonizing this pathway may impede the development of preneoplastic lesions into malignant tumors.

## Impaired mitochondrial function

**Fig. 3:**
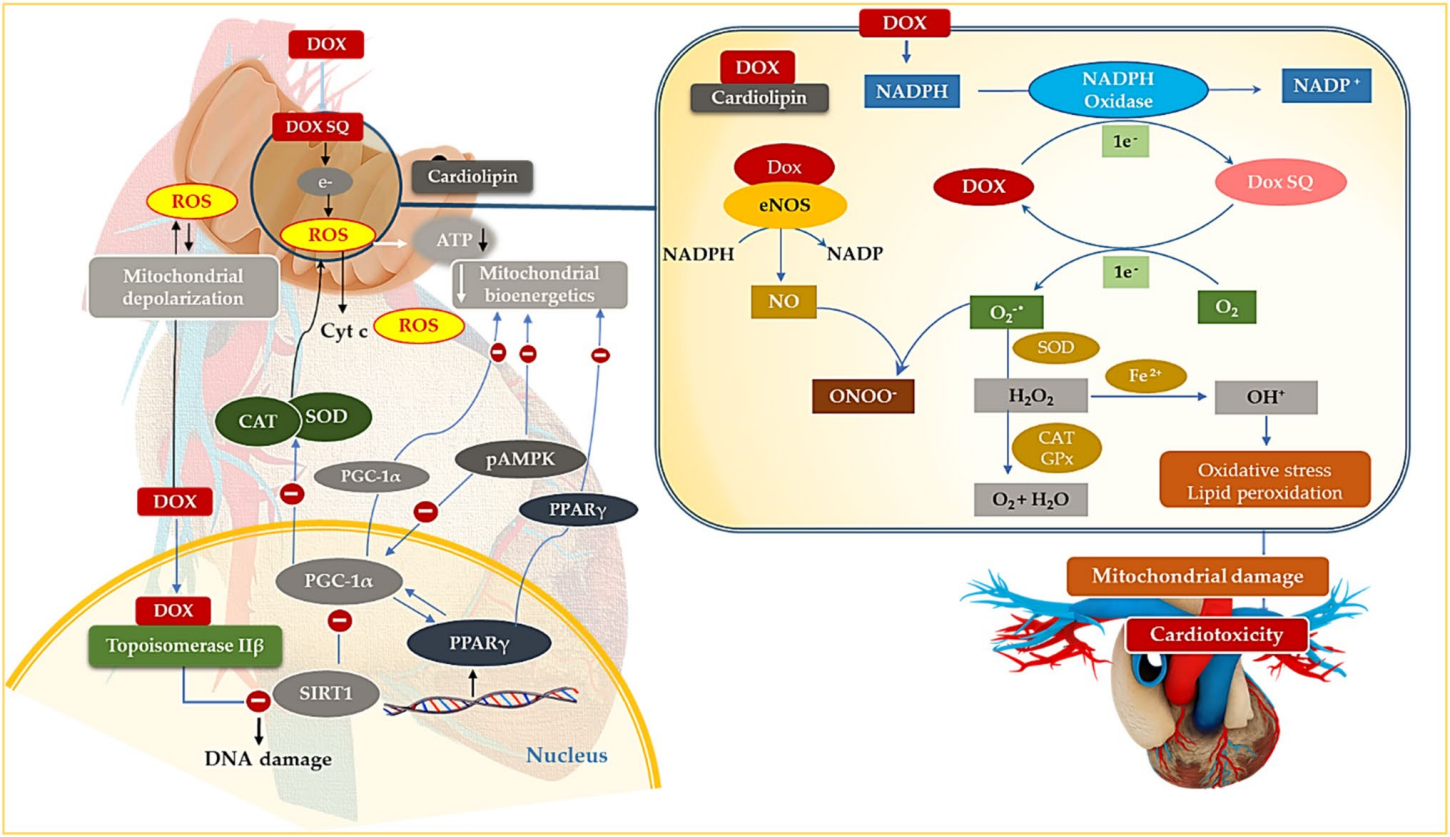
Doxorubicin-induced mitochondrial dysfunction. Concisely, NADH dehydrogenase (situated in complex I of the electron transport chain (ETC)) catalyzes the Dox redox quinone cycle to form a semiquinone. The semiquinone readily undergoes auto-oxidation by donating an electron to molecular oxygen (O_2_) to generate ROS (O^•–^_2_ and H_2_O_2_) and lipid peroxides. Increased ROS, accompanied with a paucity of antioxidants, potentiates mitochondrial-induced oxidative damage. Dox also binds to topoisomerase IIβ (Top IIβ) to induce DNA damage and the subsequent downregulation of bioenergetic genes (adenosine monophosphate-activated protein kinase (AMPK), peroxisome proliferator-activated receptor-γ (PPARγ) coactivator-1α and β (PGC-1α and β)). Impaired bioenergetics with concomitant oxidative stress disrupt the mitochondrial gradient to induce mitochondrial dysfunction

Similar to its first-generation regimens, Dox may also hinder tumor progression by triggering mitochondrial-dependent apoptosis via the dysregulation of essential mitochondrial proteins. However, due to a lack of specificity between cancer and cardiac cell lines, Dox infers the same, but more damaging, mitochondrial permeability transition in the cardiomyocytes. These findings have been replicated across numerous experimental studies (Sangweni et al. 2020; Studneva et al. 2019; Cunha-Oliveira et al. 2018). Indeed, cardiac mitochondria from Dox-treated animals presented with impaired respiratory response with no changes in the efficiency of oxidative phosphorylation, thus alluding to the propensity of Dox to accept and redirect electrons from the electron transport chain (Wallace et al. 2020; Abdullah et al. 2019) (Fig. 3). The latter is attributed to the accrual of Dox in the inner mitochondrial sheath through its binding to cardiolipin, which maintains the mitochondrial structure, function, bioenergetics, and cell survival (Wenningmann et al. 2019). Here, NADH dehydrogenase (complex I of the ETC) catalyzes the Dox redox quinone cycle to form a semiquinone. The semiquinone readily undergoes auto-oxidation by donating an electron to molecular oxygen (O_2_), converting it back to its parent compound, which forms a repeating redox cycle and so the accumulation of ROS (O^•–^_2_ and H_2_O_2_) in cardiac mitochondria (Menna et al., 2010). The apparent disruption in the transfer of electrons across the ETC, accompanied with a paucity in free radical scavenging enzymes, impairs the cells oxidative phosphorylative capacity whilst potentiating mitochondrial-induced oxidative damage (Menna et al. 2010).

Notably, Dox also alters the transcriptome via topoisomerase IIβ (Top IIβ) inhibition and by doing so impairs the expression of adenosine monophosphate-activated protein kinase (AMPK), peroxisome proliferator-activated receptor-γ (PPARγ) coactivator-1α and β (PGC-1α and β) (Wallace et al. 2020). These genes are critical regulators of mitochondrial biogenesis. The first evidence of the involvement of AMPK in DIC was demonstrated in perfused rat hearts. Here, a decrease in AMPK phosphorylation and protein levels was observed just before the onset of myocardial dysfunction, pointing to the association of impaired bioenergetics and DIC (Tokarska-Schlattner et al. 2005). In other studies, Dox treatment enhanced AMPK phosphorylation and consequently increased apoptosis, which is a crucial component in the progression of DIC (Yang et al. 2014; Chen et al. 2011). Additionally, as an upstream regulator of PGC-1α, inhibition of AMPK by Dox treatment deactivates the AMPK-PGC-1α signaling cascade, thereby impairing myocardial function (Liu et al. 2019). Literature shows that loss in PGC-1α downregulates cardiac expression of PPARγ and in turn leads to oxidative stress and metabolic dysfunction (Wang et al. 2020). Given that the complexity of PGC-1α allows it to modulate PPAR-mediated metabolic shifts whilst regulating mitochondrial proliferation makes it a suitable target for Dox-induced mitochondrial damage (Wang et al. 2020).

## Autophagy-mediated cardiotoxicity

**Fig. 4:**
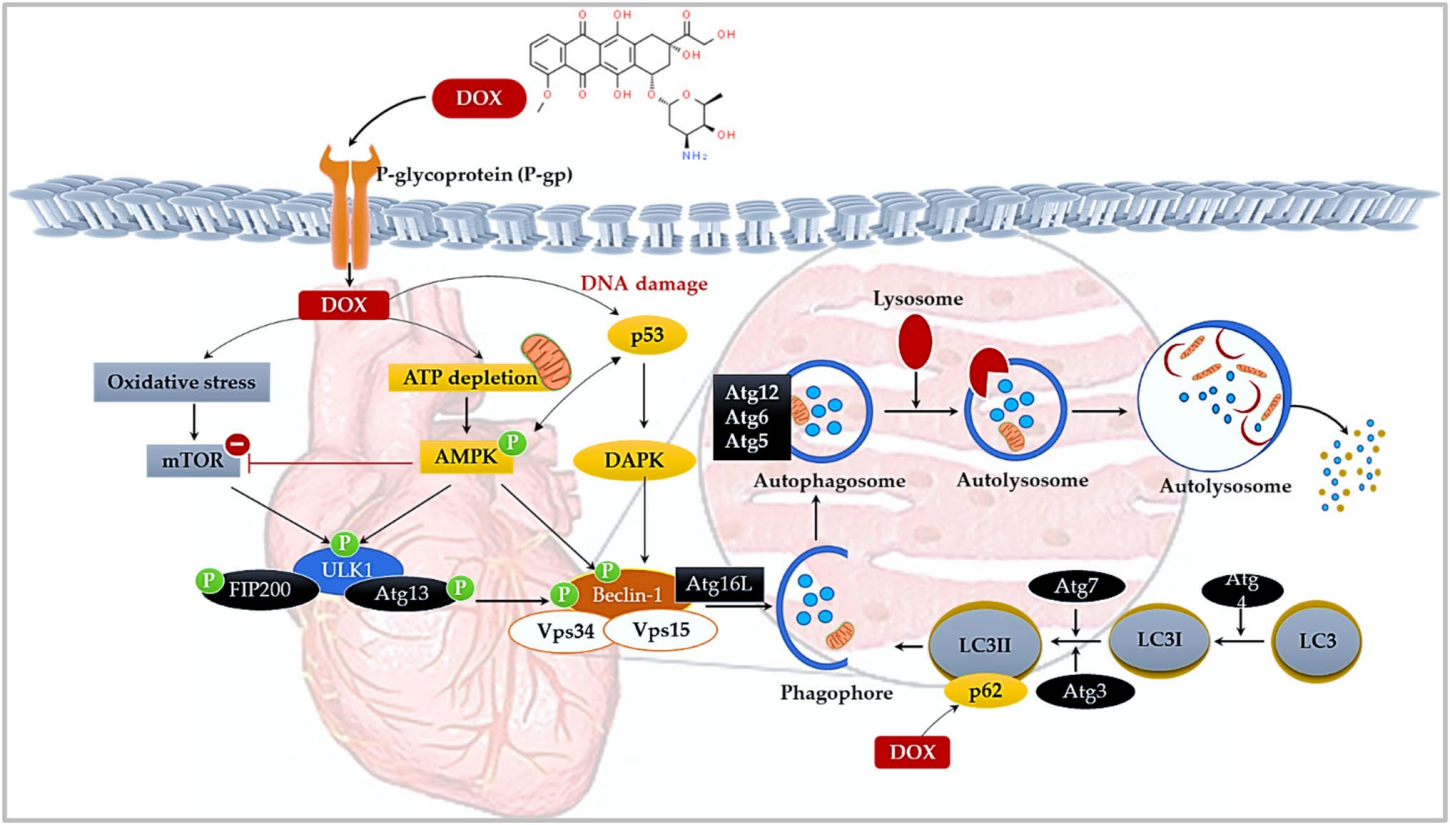
Doxorubicin-induced autophagy. The most upstream signaling protein of autophagy is regulated by AMPK and mammalian target of rapamycin (mTOR). Doxorubicin is considered a double-edged sword in autophagy, where it either inhibits or activates autophagy. Concisely, Dox inhibits the expression of mTOR whilst phosphorylating AMPK thereby, activating Beclin-1 via ULK-1. Phosphorylated Beclin-1 facilitates the assembly of the Beclin-1/ autophagy-related (Atg16L)/ vacuolar protein sorting (Vps)34/Vps15 complex, which forms autophagosomes and phagosome elongation. The light chain 3 (LC3) microtubule-associated protein enables the maturation of these phagosomes is mediated and hereafter, induces Atg4-cleavage which forms the LC3-I protein that is then lipidated by Atg 7 and 3 to LC3-II. The subsequent fusion of autophagosomes and autolysosomes is digested and removed by lysosomal enzymes

Generally, damaged mitochondria are tagged and isolated based on impaired mitochondrial membrane potential and are then engulfed by autophagosomes before being delivered to lysosomes for degradation (Thomas and Gustafsson 2013). However, this process has been shown to be disrupted by Dox administration (Russo et al. 2021). As it stands, the role of autophagy in DIC is considered a double-edged sword, as it may prevent cardiotoxicity or exacerbate the disease state if the autophagic flux surpasses a certain threshold (He et al. 2021; Johnson et al. 2017; Wang et al. 2021). The most upstream signaling protein of autophagy is modulated by the unc-51-like autophagy activating kinase 1 (ULK-1) kinase complex. This complex is tightly regulated by the AMPK and mammalian target of rapamycin (mTOR) signaling cascade, where ULK-1 is activated by AMPK and inhibited by mTOR (Koleini and Kardami 2017). However, with Dox exposure, mTOR signaling is subdued allowing the phosphorylation of Beclin-1 via ULK-1 activation. Concisely, phosphorylated Beclin-1 triggers the onset of autophagy. This process is further exacerbated by enhanced AMPK phosphorylation which continues to activate the ULK-1 complex and Beclin-1. The resultant phosphorylation of Beclin-1 facilitates the assembly of the Beclin-1/ autophagy-related (Atg16L)/ vacuolar protein sorting (Vps)34/Vps15 complex, which mediates the formation of autophagosomes and phagosome elongation, via the recruitment of more Atg proteins. Maturation of these phagosomes is mediated by the light chain 3 (LC3) microtubule-associated protein, which after Atg4-induced cleavage forms the LC3-I protein that is then lipidated by Atg 7 and 3 to LC3-II. LC3-II has a high affinity for LC3-II-interacting domain (LIR) proteins, like p62, which facilitate autophagosome degradation (Russo et al. 2021). Completion of the latter process relies on the lysosomal fusion of autophagosomes, forming autolysosomes which are digested and removed by lysosomal enzymes (Fig. 4). Paradoxically, overstimulated autophagic response appears to modulate cell death through the excessive digestion of essential cellular constituents. In this regard, autophagy levels are altered not only in response to Dox but, also to the manifesting cardiovascular dysfunction, which is in part driven by accelerated cardiomyocyte cell death (Timm and Tyler, 2020; He et al., 2021).

## Doxorubicin-mediated cell death

**Fig. 5:**
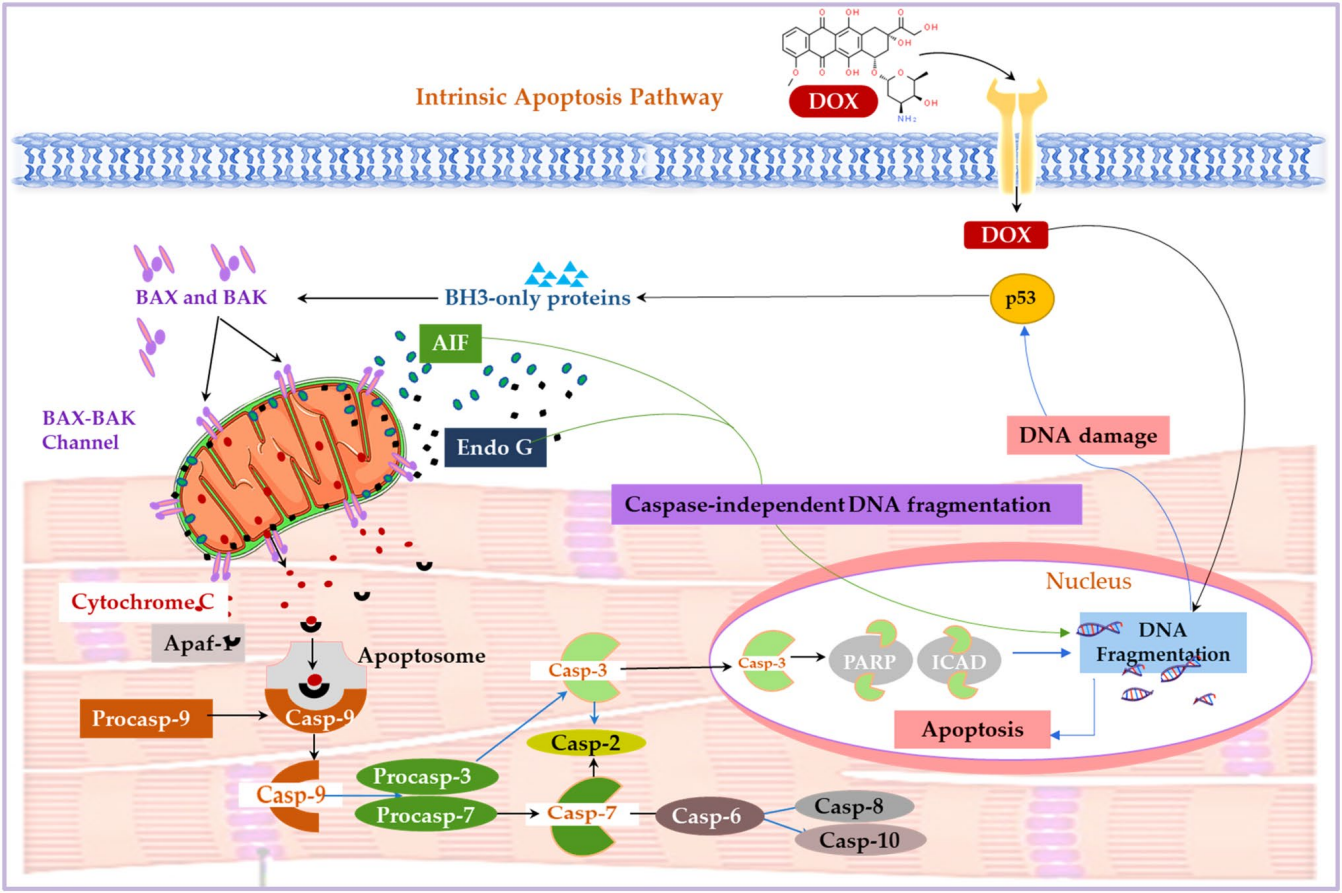
Doxorubicin-induced apoptosis via the intrinsic pathway. The release of cytochrome c, via mitochondrial dysfunction, mediates the oligomerization of apoptosis-protease activating factor-1 (Apaf-1), generating the apoptosome, which activates Casp-9 to cleave the executioner Casp-3/7. Activated Casp-3, but not Casp-7, is then translocated to the nucleus where it cleaves the inhibitor of caspase-activated DNase (CAD) to induce DNA fragmentation. Furthermore, the loss in mitochondrial potential via Dox treatment, stimulates the release of mitochondrial-bound pro-apoptotic proteins, endonuclease G (EndoG) and apoptosis-inducing factor (AIF) which are then translocated to the nucleus to induce caspase-independent DNA fragmentation and genomic instability. These processes accelerate cardiomyocyte death via dysregulated apoptosis

**Fig. 6:**
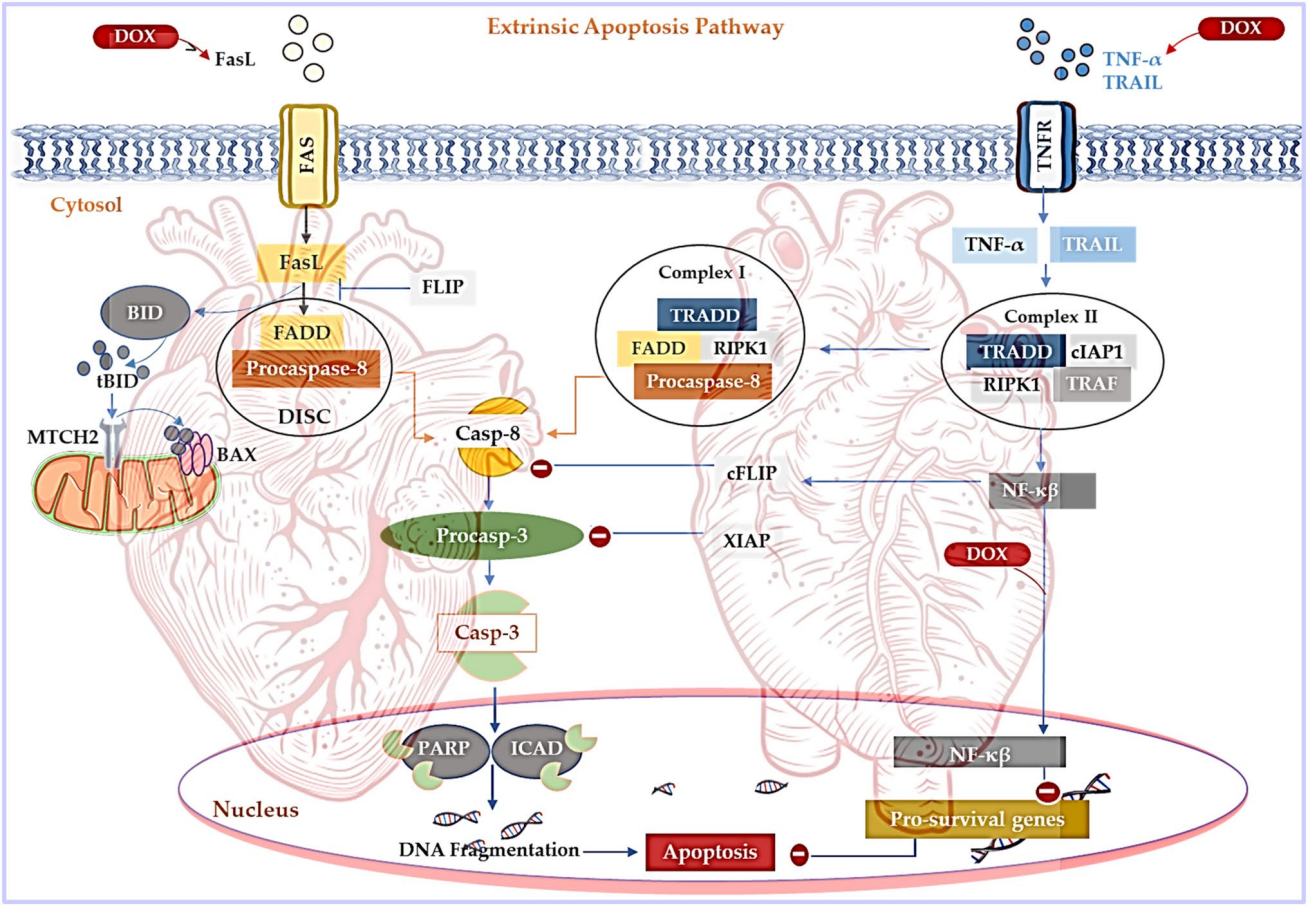
Doxorubicin-induced apoptosis via the extrinsic pathway. The extrinsic pathway is regulated by the death receptors (Fas, the TNF receptor superfamily 1A (TNFR1), TNF-related apoptosis-inducing ligand receptors 10a (TRAIL1) and TRAIL2 (10b)) which are overstimulated during Dox exposure. The death-inducing signaling complex (DISC), “complexes I and II”, is formulated by the assembly of dynamic multi-proteins, that operate as molecular platforms that regulate Casp-8. Activated Casp-8 facilitates the proteolytic maturation of executioner Casp-3/6/7 which drives apoptosis via DNA damage. Simultaneously, Dox-mediated cleavage of pro-apoptotic BH3-interacting domain death agonist (BID) forms truncated BID (tBID). The tBID is then translocated to the outer mitochondrial membrane via a Fas-dependent pathway, which is reliant on the binding of modulator of apoptosis 1 (MOAP1) to the BID receptor mitochondrial carrier 2 (MTCH2). Dox also mediates the reduction of cFLIPs and XIAP via NF-κβ overexpression to trigger the onset and progression of apoptosis

Although numerous cell death pathways are intricately involved in the onset and progression of DIC, the most well recognized and studied is apoptosis, which is an automated process involving the disposal of cells with little or no inflammation occurring in surrounding tissues. In the physiologically state, apoptosis regulates the development of cardiomyocytes and cardiac homeostasis (Loreto et al. 2014). However, dysregulated apoptosis is a key player in cardiac remodeling and the subsequent development of LVD, which is a hallmark of DIC (Lipshultz et al., 2013). The two canonical signaling pathways that drive apoptosis are the intrinsic and extrinsic pathways, which are elaborated below (Figs. 5 and 6).

### Intrinsic apoptotic pathway

The activation of the intrinsic pathway, via Dox administration, requires mitochondrial outer membrane permeabilization (MOMP), an event that is coined the 'point of no return' during apoptosis. Briefly, permeabilized mitochondria release cytochrome c (Cyt c), which is a peripheral intra-mitochondrial protein that shuttles electrons from complex III and IV (Loreto et al. 2014). Upon its release into the cytosol, Cyt c mediates the oligomerization of apoptosis-protease activating factor-1 (Apaf-1), generating the apoptosome that recruits inactive dimers of Casp-9. The apoptosome cleaves and activates Casp-9 by hydrolyzing ATP, which then activates and cleaves the executioner Casp-3/7. Activated Casp-3, but not Casp-7, is then translocated from the cytosol to the nucleus to induce apoptosis. Once in the nucleus, Casp-3 stimulates the release and activation of caspase-activated DNase (CAD) by cleaving the inhibitor of CAD (ICAD) (Kitazumi and Tsukahara 2011), which facilitates the fragmentation of DNA. Although Casp-7 does not translocate to the nucleus, it is still able to cleave ICAD but, less efficiently than Casp-3 (Walsh et al. 2008). In response to DNA damage, poly(ADP-ribose) polymerase (PARP) is activated to enable DNA repair and genomic stability. However, Dox-mediated Casp-3 activation inhibits PARP activity and inactivates its DNA repairing abilities during apoptosis. (Park et al. 2018). Conversely, the induction of DNA fragmentation can also be initiated in a caspase-independent manner. The loss in mitochondrial membrane potential, by Dox, stimulates the cleavage and release of mitochondrial-bound pro-apoptotic proteins, endonuclease G (EndoG) and apoptosis-inducing factor (AIF) which are translocated to the nucleus. EndoG, a double and single-stranded DNase and RNase, triggers the cleavage of higher-order chromatin into HRMM DNA fragments (over 50 kb) and the formation of numerous single-stranded nicks (190 bp at nucleosomal and 10 bp at subnucleosomal periodicities) through intra- and inter-nucleosomal DNA cleavage (Kitazumi and Tsukahara 2011). Although AIF is a mitochondrial flavoprotein without DNase activity that initiates chromatin condensation and DNA cleavage into HRMM fragments, it is unable to stimulate oligonuclesomal DNA fragmentations (Artus et al. 2010) (Fig. 5).

### Extrinsic apoptotic pathway

On the other hand, Dox-induced apoptosis via the extrinsic pathway is driven by the death receptors, which rely on the binding of cognate ligands (Galluzzi et al. 2018) (Fig. 6). The death receptors consist of Fas, the TNF receptor superfamily 1A (TNFR1), TNF-related apoptosis-inducing ligand receptors 10a (TRAIL1) and TRAIL2 (10b). While cardiac cells are often resistant to Fas-mediated apoptosis, literature shows that Dox-induced apoptosis in the myocardium can be executed through the Fas pathway (Font-Belmonte et al. 2019; Galluzzi et al. 2018). The death-inducing signaling complex (DISC), “complex I and II”, is formulated by the assembly of dynamic multi-proteins, that operate as molecular platforms that regulate Casp-8. Maturation of Casp-8 involves a cascade of events triggered by the binding of FADD to Casp-8 at the DISC, which facilitates the homodimerization and the resultant activation of Casp-8 by autoproteolytic cleavage. In type I cells such as lymphocytes, apoptosis is initiated by the proteolytic maturation of executioner Casp-3/7 through Casp-8 activation. Once initiated, this process cannot be subdued even by the overexpression of the anti-apoptotic BCL-2 protein or the deletion of pro-apoptotic Bax and Bak1 (Galluzzi et al. 2018). In contrast, apoptosis in type II cells, like cardiomyocytes, requires the proteolytic cleavage of pro-apoptotic BH3-interacting domain death agonist (BID) by Casp-8, which formulates a truncated form of BID (tBID). The tBID is then translocated to the outer mitochondrial membrane via a Fas-dependent pathway, which is reliant on the binding of modulator of apoptosis 1 (MOAP1) to the BID receptor mitochondrial carrier 2 (MTCH2) (Galluzzi et al. 2018). In the same context, Casp-3/7 activation in these type II cells is inhibited by X-linked inhibitor of apoptosis (XIAP). However, Dox administration has been shown to mediate the reduction of cFLIPs and XIAP through overexpression of NF-κβ whilst enhancing the expression of TRAIL to trigger the onset and progression of apoptosis (Fig. 6).

## Conclusion

It is quite evident that the more we understand about the clinical implications and the basic mechanisms that have been identified to be associated with DIC, the better our understanding will be on the cardiotoxicity that ensues years after treatment cessation. This is especially important in cardio-oncology research to identify alternative and novel therapeutics to delay or prevent the occurrence of cardiovascular complications.
